# Double fluorescence-guided surgery with 5-ALA and fluorescein sodium in grade 2 and grade 3 adult-type diffuse gliomas: retrospective analysis of 112 cases

**DOI:** 10.1016/j.bas.2025.104277

**Published:** 2025-05-09

**Authors:** Andrea Bianconi, Marta Bonada, Pietro Zeppa, Francesco Bruno, Pietro La Cava, Flavio Panico, Roberta Rudà, Antonio Melcarne, Diego Garbossa, Fabio Cofano

**Affiliations:** a Division of Neurosurgery, “Città della Salute e della Scienza” University Hospital, Department of Neuroscience “Rita Levi Montalcini”, University of Turin, 10124, Turin, Italy; b Division of Neuro-Oncology, “Città della Salute e della Scienza” University Hospital, Department of Neuroscience “Rita Levi Montalcini”, University of Turin, 10124, Turin, Italy

**Keywords:** Glioma, Low grade, Fluorescence guided, Fluorescein, 5-ALA, Aminolevulinic acid

## Abstract

**Objective:**

Fluorescence-guided surgery (FGS) has been increasingly used to support glioma surgery to obtain a maximal extent of resection (EOR). Current evidence in lower-grade gliomas does not support the routine use of FGS obtained with the most common fluorescence agents (e.g. 5-ALA and fluorescein sodium). However, the combination of these two dyes has not been extensively explored yet. Main objective of this study is to evaluate the role of 5-ALA and FS in LGGs surgery for tumor detection, margin definition, and prognostic relevance.

**Methods:**

112 patients affected by a histologically confirmed adult-type diffuse glioma grade 2–3 molecularly defined underwent craniotomy in “Città della Salute e della Scienza” hospital (Turin, Italy). Surgery has been performed under general anesthesia with the previous administration of both 5-ALA (20 mg/kg) and fluorescein sodium (3 mg/kg). We retrospectively investigated clinical, radiological, histological and molecular data. Fluorescence positive rate and pattern have been reported both for 5-ALA and for fluoresceine.

**Results:**

We included 69 patients with astrocytoma *IDH-*mutant and 43 with oligodendroglioma *IDH-*mutant 1p19q-codeleted. Seventeen cases were positive for both 5-ALA and FS (15.1 %), 24 for 5-ALA (21.4 %) only, 1 for FS (1.0 %) only, 70 were negative (62.5 %). The relationship between intraoperative fluorescence and the presence of foci with contrast enhancement uptake on the preoperative MRI was statistically significant (p < 0.001) for both the dyes. 5-ALA intraoperative detection had a statistically significant impact on the overall survival (OS) (HR: 2.51, 95 % CI: 1.25–5.01, p = 0.009) and progression-free survival (PFS) (HR: 2.46, 95 % CI: 1.34–4.52, p = 0.004). Additionally, both FS and 5-ALA fluorescence slightly prevailed in grade 3 gliomas, especially 5-ALA.

**Conclusion:**

The results achieved in this study do not support the role of 5-ALA and FS to intraoperatively define the extent of resection, because of low fluorescence rates. Nevertheless, 5-ALA expression could be used to intraoperatively identify more aggressive foci and add useful prognostic information before the histological analysis. Indeed, FS is mostly related to blood-brain barrier damage and, thus, with contrast enhancement in MRI.

## Highlights

• The absolute prevalence of fluorescence positivity was 36.6 % for 5-ALA and 16 % for sodium fluorescein.
• Both 5-ALA and SF fluorescence were more frequently associated with grade 3 gliomas compared to grade 2 gliomas.
• Fluorescence expression, particularly from 5-ALA, was associated with shorter overall survival (OS) and progression-free survival (PFS).

## 1 Introduction

Extent of resection (EOR) is associated with increased overall survival (OS) in both low-grade and high-grade gliomas (Cofano et al., 2024; Hervey-Jumper et al., 2023; Karschnia et al., 2024b; Morshed et al., 2019). The main technical tools used to improve the EOR include neuronavigation, intraoperative magnetic resonance imaging (MRI), intraoperative ultrasound and fluorescence-guided surgery (FGS). (Almekkawi et al., 2020; Molina et al., 2018; Roder et al., 2017) These techniques are particularly useful in glioma surgery, supporting the intraoperative distinction between normal and pathological tissue (Hadjipanayis et al., 2015). Particularly, FGS is considered as an additional strategy to identify intraoperatively the tumor, as neoplastic cells have a higher fluorophore uptake compared to normal brain tissue (Hendricks et al., 2019; Li et al., 2014).

Currently, the most commonly used fluorescent-labeling dye is 5-aminolevulinic acid (5-ALA), a natural metabolite of the heme synthesis pathway (Belykh et al., 2016). For glioblastoma (GBM), the use of 5-ALA to guide tumor resection has been proven to increase the EOR and prolong overall survival (OS) and progression-free survival (PFS) (Aldave et al., 2013; Lau et al., 2016; Picart et al., 2023; Schatlo et al., 2015). Other fluorescence agents have raised interest for their characteristics and their potential use in glioma surgery (Senders et al., 2017). Among these, the most investigated is fluorescein sodium (FS), which does not accumulate selectively in glioma cells, rather being sensitive to blood-brain barrier damage (Acerbi et al., 2018; Zeppa et al., 2022). Given its properties, it locates in areas with high tumoral cell density and fast proliferation, thus helping the recognition of enhancing tumor regions. While 5-ALA is currently the only approved drug for fluorescence-guided surger in glioma resections, recent studies indicate that FS offers several advantages over 5-ALA, including broader indications, affordability, lack of toxicity, and simpler intraoperative administration (Pesaresi et al., 2024). Other agents have been proposed, yet not extensively assessed, such as indocyanine green (ICG), hypericin, 5-aminofluorescein-human serum albumin, BLZ-100 (Tozuleristide) (Bianconi et al., 2023). Differently, some studies are focused on new detection modalities to recognize the presence of fluorophores at concentrations not perceptible by the surgeon's vision (Belykh et al., 2018). These strategies include confocal laser endomicroscopy (CLE) and laser spectroscopy (Valdés et al., 2015; Wei et al., 2019).

Concerning grade 2–3 gliomas, there is no clear advantage in the routine use of these fluorophore with a standard operating microscope, with the aim of defining the tumor margin (Bianconi et al., 2023; Hendricks et al., 2019). Anyway, the literature is still limited on this topic, and the available results are not easily comparable. Thus, the possible benefits of FGS for these tumors needs to be further investigated and established (Jaber et al., 2016; Nishikawa, 2011). Many possible strategies may provide a solution to increase the accuracy of tumor intraoperative detection, such as the combinatory use of available fluorophores, the application of new detection instruments, the study of new fluorescent dyes. To explore these different possible strategies would help to address research efforts in the right direction and exclude the ones that do not provide an accurate support for intraoperative tumor detection.

The main purpose of this study was to report a single-center experience with the use of both 5-ALA and FS in grade 2-3 adult-type diffuse gliomas. In particular, the aim was to explore the use of these dyes to estimate their accuracy in tumor detection, define tumor boundaries, and assess their prognostic value, suggesting their possible future role in FGS for this type of tumors.

## 2 Methods

### 2.1 patient selection

A single-center retrospective analysis in “Azienda Ospedaliera Universitaria Città della Salute e della Scienza”, University Hospital of Turin (Italy) was conducted. All patients who underwent craniotomy for an intra-axial brain tumors were considered recruitable. Inclusion criteria were the availability of all clinical and surgical records, preoperative and postoperative MRI imaging, use of both the fluorescent dyes (5-ALA and FS), and integrated histological and molecular diagnosis of a diffuse glioma grade 2 or grade 3 (according to WHO, 2016 and 2021 depending on the date of surgery) (Louis et al., 2021). The exclusion criteria were: (1) a histological diagnosis other than glioma grade 2–3; (2) a tumor that originated in the midline, basal ganglia, cerebellum, or brainstem; (3) multicentric tumors; (4) medical reasons precluding MRI (for example, the presence of a pacemaker); (5) inability to give consent because of dysphasia or language barrier; (6) a preoperative Karnofsky Performance Status (KPS) score of 60 or less; (7) a history of active malignant tumors at any other site. All procedures performed for this study were in accordance with the ethical standards of “Azienda Ospedaliera Universitaria Città della Salute e della Scienza”, University Hospital, University of Turin and with the 1964 Helsinki declaration and its later amendments or comparable ethical standards.

### 2.2 Surgical strategy

Regarding fluorescent dyes: 5 ALA (20 mg/kg) alone was administered orally 2.5–3.5 h before anesthesia induction, and FS (3 mg/kg) was administered intravenously at the anesthesia induction. All patients were treated to achieve a maximal safe resection of the radiologically recognizable tumor volume. A Leica M530 OHX (Leica Microsystems, Heerbrugg, Switzerland), equipped with both FL 400 and FL 560 filters to emit and observe different wavelength ranges, was used interchangeably to detect 5-ALA and FS, respectively. All the procedures were conducted with the aid of a neuronavigation system (Brainlab iPlan 3.0 and Elements, BrainLAB AG, Munich, Germany or Medtronic systems Stealth Station S7, Medtronic Inc., Dublin, Ireland). Surgical strategy contemplated a first white light inspection of the tumor and a subsequent analysis of the fluorescent pattern before the excision. This evaluation was carried out intraoperatively and established by surgical operators in agreement. A postoperative contrast-enhanced brain MRI was performed within 48 h from the surgery. EOR was defined according to Karschnia et al. (2021). A volumetric analysis was performed on preoperative and postoperative images through manual segmentation (Horos Project, www.horosproject.org), as previously described. One month after surgery, all patients were evaluated in a multidisciplinary context to define the best treatment for the patient according to current guidelines.

### 2.3 Statistical analysis

Data are expressed as mean (±standard deviation) for continuous variables, as frequencies and percentages for categorical data. Outcome variables were compared using the χ 2 test and Fisher's exact test for categorical variables. A Kaplan-Meier analysis and log-rank test were used to compare OS between groups. Statistical significance level was set at p < 0.05. All statistical analyses were performed using Jamovi software version 2.2 (The jamovi project (2021). Retrieved from https://www.jamovi.org.)

## 3 Results

The study included 112 patients operated for a grade 2 or grade 3 adult-type diffuse glioma according to WHO classification 2016 and 2021. Patients’ characteristics are described in Table 1.

**Table 1:** Population characteristics.

Population Characteristics	N = 112
Sex: male	52 (46 %)
Age (years)	48.7 (19–76)
Seizure at onset: yes	50 (45 %)
Lobe	
Precentral	49 (44 %)
Postcentral	31 (27 %)
Temporo-Insular	32 (29 %)
Side: left	55 (49 %)
MRI Contrast enhancement: yes	15 (13 %)
Histology	
Astrocytoma	69 (62 %)
Oligodendroglioma	43 (38 %)
WHO Grade: 2	52 (46 %)
MGMT methylation: yes	46 (41 %)
IDH1 Mutation: yes	102 (91 %)
1p 19q Codeletion: yes	43 (38 %)
Extent of resection:	
Supratotal	4 (4 %)
Gross total	80 (71 %)
Subtotal	19 (17 %)
Partial	9 (8 %)
KPS (1 month after surgery), median	90
Adjuvant radiotherapy: yes	52 (46 %)
Adjuvant Temozolomide: yes	64 (57 %)
Progression Free Survival (months)	24.9 ± 25.6
Overall Survival (months)	38.0 ± 28.6

Specifically, histological examination after surgery demonstrated 52 cases of grade 2 and 60 cases of grade 3 gliomas. 69 cases were classified as astrocytoma *IDH*-mutant, (28 grade 2 and 41 grade 3), whereas 43 cases were classified as oligodendroglioma *IDH*-mutant, 1p19q-codeleted including 24 grade 2 and 19 grade 3 tumors.

Fluorescence positivity for each dye has been assessed intraoperatively, resulting in 17 cases classified as positive for both 5-ALA and FS (15.1 %), 24 only for 5-ALA (21.4 %), 1 only for FS (1.0 %), 70 negative cases (62.5 %). Specifically, 5-ALA was positively recognized intraoperatively in 41 cases (35.9 %) while FS in 18 cases (16.2 %) (Table 2).

**Table 2:** Fluorescence distribution according to tumor subtypes.

		None	5-ALA	FS	Both	
All		70 (62.5 %)	41 (36.6 %)	18 (16.0 %)	17 (15.1 %)	
Histology	Oligodendroglioma	25 (22.3 %)	17 (15.1 %)	7 (6.2 %)	6 (5.4 %)	X^2^ = 2.45 P = 0.48
	Astrocytoma	45 (40.2 %)	24 (21.4 %)	11 (9.8 %)	11 (9.8 %)	
Grade	2	37 (33.0 %)	15 (13.4 %)	6 (5.3 %)	6 (5.4 %)	X^2^ = 3.65 p = 0.30
	3	33 (29.5 %)	26 (23.2 %)	12 (10.7 %)	11 (9.8 %)	
IDH mutation	yes	63 (56.3 %)	38 (33.9 %)	17 (15.1 %)	16 (14.3 %)	X^2^ = 0.401 p = 0.940
	no	7 (6.3 %)	3 (2.6 %)	1 (0.8 %)	1 (0,9 %)	
MGMT methylation	yes	24 (21.4 %)	18 (16.0 %)	8 (7.1 %)	7 (6.2 %)	X^2^ = 2.72 p = 0.43
	no	46 (41.0 %)	23 (20.5 %)	10 (8.9 %)	10 (8.9 %)	
MRI Contrast enhancement	yes	1 (0,9 %)	13 (11.6 %)	13 (11.6 %)	12 (10.7 %)	X^2^ = 64.8 p < 0.001
	no	69 (61.6 %)	28 (25.0 %)	5 (4.5 %)	5 (4.5 %)	

**Fig. 1:**
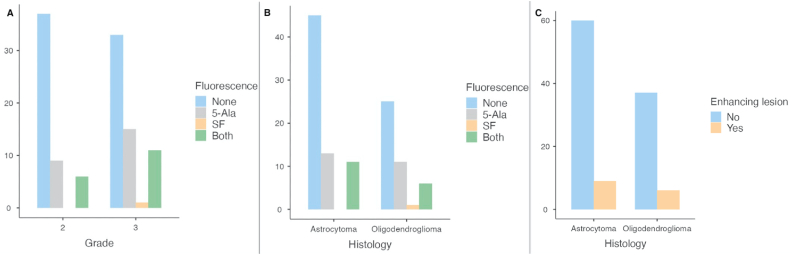
Graphs showing the relationship between fluorescence and tumor grade (A), histology (B), MGMT methylation profile (C).

The relationship between grade and fluorescence positive expression has been evaluated (Fig. 1a). Both FS and 5-ALA expression prevailed in grade 3 gliomas. In particular, FS was positive in 6/52 grade 2 gliomas (11.5 %) and 12/52 grade 3 gliomas (23.1 %) (p = 0.224); 5-ALA was positive in 15/60 grade 2 gliomas (25.0 %) and 26/60 grade 3 gliomas (43.3 %) (p = 0.112). No statistically significant association was found between IDH mutation and fluorescence with either fluorophore.

FS was positive for 11/69 cases of astrocytoma (16.0 %) and 7/43 cases of oligodendroglioma (16.3 %); 5-ALA was positive for 24/69 cases of astrocytoma (34.8 %) and 17/43 cases of oligodendroglioma (39.5 %), without significant correlation among histological subtypes (Fig. 1b). Also molecular data have been considered, including MGMT promotor methylation status. In particular, MGMT promotor was methylated for 7/17 (41.1 %) cases positive to both 5-ALA and FS, for 11/24 (45.8 %) cases exclusively positive to 5-ALA, for 1/1 (100 %) case exclusively positive to FS and for 24/70 (34.3 %) cases negative to both the dyes (Fig. 1c). The relationship between MGMT status and fluorescence uptake was not statistically significant (p = 0.437).

The relationship between intraoperative fluorescence and the presence of foci with contrast enhancement uptake on the preoperative MRI (pMRI) has been evaluated. 15 cases presented contrast enhancement (CE) on the pMRI (10.7 %). Among these cases, 12 were positive to both 5-ALA and FS (80.0 %), 1 was exclusively positive to FS (8.3 %) and 1 to 5-ALA (8.3 %), and the one remaining case was negative for the intra-operative fluorescence (Table 2). The relationship between CE on pMRI and fluorescence positive expression was statistically significant (p < 0.001) for both the combination (5-ALA and FS) and the single positivity to one of the dyes (5-ALA or FS). Notably, among the 18 cases positive for FS, 13 presented foci of CE in preoperative MRI (72.2 %). Also, for 5-ALA the relationship with pMRI CE was statistically significant (p < 0.001). Considering tumor grade, 5/52 (9.6 %) grade 2 and 10/60 (16.7 %) grade 3 had CE. Thus, even if grade 3 were more frequently presenting positive contrast uptake, the result is not statistically relevant (p = 0.275). After multivariate analysis, CE on pMRI remained the only statistically significant variable (p < 0.001), independently of histology, grade, and molecular status. No other variables reached statistical significance.

**Fig. 2:**
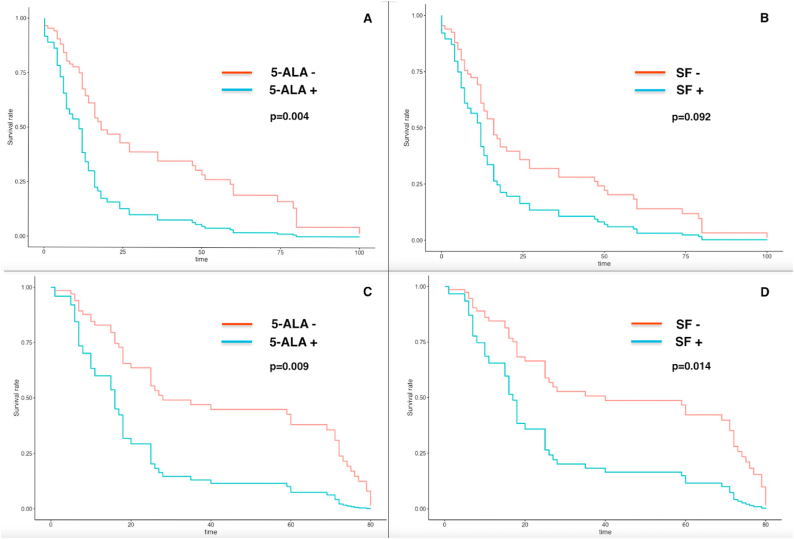
Kaplan-Meier survival curves adjusted for tumor grade, for (A) PFS in 5-ALA positive patients (B) PFS in FS positive patients, (C) OS in 5-ALA positive patients, (D) OS in FS positive patients.

All patients underwent a clinical and radiological follow-up to evaluate OS and PFS and their relationship with 5-ALA and FS positivity. We performed both univariate and multivariate analysis, thus eliminating possible interferences by age, performance status, tumor grade, preoperative contrast uptake on MRI, extent of resection. FS positive cases were related to lower OS and PFS, with a statistically significant result for OS (HR: 2.72, CI: 1.22–6.06, p = 0.014) but not for PFS (HR: 1.77, CI: 0.91–3.43, p = 0.092). For 5-ALA expression, lower OS (17,5 vs. 37,5 months) and PFS (39.3 vs. 60.7 months) emerged from the analysis. The results were statistically significant for both OS (HR: 2.51, CI: 1.25–5.01, p = 0.009) and PFS (HR: 2.46, CI: 1.34–4.52, p = 0.004). In the multivariate analysis, tumor grade did not significantly influence the previous results, concerning both PFS (p = 0.369) and OS (p = 0.833). The same could be reported for contrast uptake on preoperative MRI (Fig. 2).

Additionally, we decided to considerate separately grade 2 and grade 3 tumors, to observe eventual differences. Considering exclusively grade 2 tumors, FS positivity was not related to lower PFS (13 vs. 16 months, p = 0.457) and lower OS (18 vs. 71 months, p = 0.167), while regarding 5-ALA, the uptake was significantly associated with a worsening of both PFS (18 vs. 71 months, p = 0.02) and OS (18 vs. 71 months, p = 0.01). Similarly for grade 3 lesions, the results concerning FS positivity showed a non-significant trend towards lower PFS (12 vs. 16 months, p = 0.165) and lower OS (17 vs. 25 months, p = 0.061). 5-ALA positivity was related to lower PFS (12 vs. 18 months, p = 0.051) and OS (17 vs. 27 months, p = 0.019).

## 4 Discussion

In our single-center sample of patients newly diagnosed with grade 2–3 glioma, our main findings showed that the absolute prevalence of fluorescence positivity was 36.6 % for 5-ALA and 16 % for FS, with an association with the presence of contrast enhancement foci on MRI, but without an association with histological grade, IDH mutation, or MGMT methylation. Additionally, 5-ALA and FS fluorescence were associated with shorter OS and PFS.

Nowadays, the advantage of the routine use of both fluorophores has not yet been established for grade 2 and 3 gliomas because of low fluorescence rates, although data are still limited on this topic and the available results are not easily comparable (Bianconi et al., 2023; Hendricks et al., 2019). For this reason, it is essential to define the possible benefits of FGS with currently available fluorescent dyes and techniques before the investigation of alternative methods (Jaber et al., 2016; Nishikawa, 2011).

Grade 2 and grade 3 can present with similar clinical and radiological characteristics, despite different histological-molecular features. Moreover, it is now well established that grade 2 and 3 gliomas are a spectrum of tumors more than separate entities (Picca et al., 2023). Even if a conclusive diagnosis cannot be achieved until a histological and molecular analysis is carried out, some radiological features can be helpful to infer tumor grade (De Marco et al., 2022). For instance, the presence of contrast enhancement on the pMRI may support the presence of high-grade areas. In fact, even if the absence of CE does not allow to exclude a grade 3 glioma or even a glioblastoma, and CE foci on MRI could rarely be observed also in grade 2 gliomas (especially oligodendrogliomas), the presence of contrast-enhanced areas in LGGs usually correspond to regions of higher proliferation index and/or higher grade (Karschnia et al., 2024a; Nakamoto et al., 2019; Specchia et al., 2021). Interestingly, in our study the presence of CE on the pMRI was significantly associated with a positive fluorescence expression, which was confirmed for both the combination (5-ALA and FS) and the single positivity of one of the two dyes (5-ALA or FS). In this context, the finding of any fluorescence, in spots or diffusely, might lead neurosurgeons to redefine their intraoperative goals, targeting the tissue sampling based on the real-time intraoperative findings more easily than by performing multiple frozen section biopsies. Having the most relevant tissue for tumor grading.

**Fig. 3:**
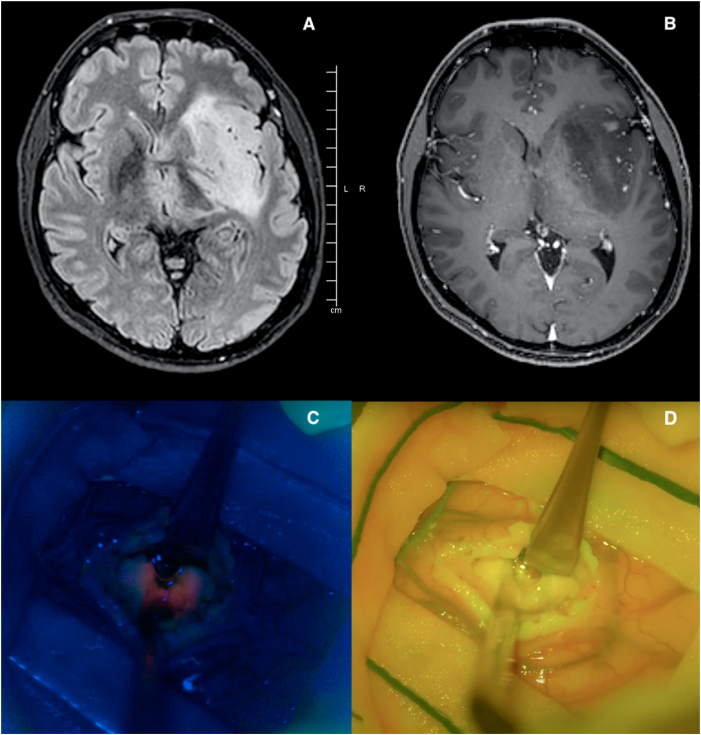
the histological diagnosis was astrocytoma grade 2 (according to WHO, 2021 classification); preoperative MRI with T2-FLAIR (A) and T1ce (B) sequences showing a spot of contrast enhancement; intraoperative image at optical microscope with FL 400 (C) showing 5-ALA positivity in one region of the tumor, and with FL 560 (D) showing the absence of FS uptake.

An illustrative case is shown in Fig. 3, where a grade 2 glioma with a spot of contrast-enhancement shows a selective 5-ALA uptake in a small portion of the tumor.

This was already reported by other groups, such as in the study from Jaber et al. (2022), that developed a model for predicting fluorescence and tumor grade considering 14 clinical and molecular variables.

The results obtained in this monocentric study including 121 patients are in accord to previous studies for fluorescence positive rates. In particular, FS rates were 11.5 % and 23.1 % while 5-ALA rates were 25.0 % and 43.3 %, respectively for grade 2 and 3 tumors. Thus, these data confirm that the detection rate is not sufficient to rely on FGS for intraoperative tumor border definition. Concerning FS, fluorescence rates are lower than 5-ALA both in this study and in the literature (Schebesch et al., 2018; Xiang et al., 2018). Differently to 5-ALA, FS is not a metabolic agent and, thus, it does not selectively accumulate in glioma cells. Indeed, as a marker of blood-brain barrier (BBB) disruption, it is more commonly present in GBM than in LGGs (Save et al., 2019). However, FS is still considered as a potential candidate to improve glioma surgery outcomes, and further studies should explore new strategies to obtain higher fluorescence rates (Bianconi et al., 2023). A randomized trial by Chen et al. observed significant differences in GTR rates (*p* = 0.047) and PFS (*p* = 0.033), with better results obtained for the group that received high doses of FS(Chen et al., 2012). Anyway, the trial included only 22 patients and the control group did not receive FS. This strategy should be further explored in this field, to verify the possible advantages and adverse events related to higher doses of FS.

5-ALA obtained higher fluorescence rate, but still under 50 % in our cases and in the literature. A possible way to explore is the optimization of surgery according to time kinetics of this fluorophore. A recent ex vivo study by Kaneko et al. highlighted that the highest fluorescence intensity and PpIX concentration can be observed 7–8 h after 5-ALA administration (Kaneko et al., 2022). Thus, different timing for administration might improve fluorescence visualization, especially in weakly fluorescing tumors (Almekkawi et al., 2020; Kaneko et al., 2022). Additionally, augmentation techniques provided promising results in 5-ALA intraoperative visualization. In fact, it is hypnotized that fluorescence may not be completely absent, despite remaining below the detection threshold for standard operating microscopes (Hendricks et al., 2019; Wei et al., 2019).

In particular, CLE and laser spectroscopic detection have already been proposed and explored by some groups with interesting results. These advanced intraoperative techniques offer increased sensitivity for detecting microscopic tumor infiltration at the margins: even if they use the 5-ALA and FS, they are more similar in their rationale of use to intraoperative histopathological frozen section analysis, as they rely on dedicated probes applied to specific points rather than providing a global overview of the surgical field through the operating microscope itself (Abramov et al., 2025; Lechpammer, 2024; Wagner et al., 2024).However they require additional instrumentation, incur higher costs, and are not widely available in all neurosurgical centers (Belykh et al., 2019, 2021; Iturrioz et al., 2022; Martirosyan et al., 2014). Moreover, these tools are generally more time-consuming during surgery compared to standard operating microscope, even if they are time sparing compared to classic frozen section analysis (Wagner et al., 2024).

In particular, quantitative spectroscopy allows the detection of minimal, non-visible amounts of 5-ALA-induced protoporphyrin IX, showing strong potential for identifying LGGs. These tumors exhibit a distinct spectroscopic pattern that helps differentiate them from both normal brain tissue and high-grade gliomas (Picart et al., 2024).Although CLE with FS is a promising tool, its performance in LGGs still falls short of standard intraoperative histology. Overall, these technologies may enhance—but not yet replace—conventional histopathological methods in LGG management (Lechpammer, 2024).

While the same fluorophores are used, the rationale and method of application differ significantly: approaches such as CLE focus on detecting microscopic infiltration at the tumor margins, whereas in our study, FS and 5-ALA are applied using standard operating microscopy resulting in highlighting biologically aggressive foci within the tumor.

The available studies focus their attention especially on positive fluorescence rates, but it is important to recognize that, in LGGs, the primary goal of FGS is not to delineate tumor margins. For this reason, we decided to explore the relationship between fluorescence intraoperative detection and other variables, such as prognostic information and tumor histological and molecular features. Concerning histological features, gliomas are known to demonstrate great heterogeneity within the same tumoral lesion. Thus, also PpIX fluorescent foci distribution is hypothesized to represent neoplastic regions with higher rates of cell proliferation and a greater propensity for malignant transformation (Lau et al., 2016; Widhalm et al., 2010). More specifically, visible 5-ALA fluorescence was hypothesized to be related with histopathological criteria of malignancy, such as the mitotic rate and cell density, thus representing an indicator of aggressive tumor behavior (Jaber et al., 2019). However, despite the growing body of evidence linking intraoperative fluorescence with higher tumour grade and markers of aggressiveness, the relationship is not always straightforward, and fluorescence cannot be considered a universally reliable indicator in lower-grade gliomas. For example, Hosmann et al. reported a subset of histologically confirmed grade 3 gliomas exhibited no intraoperative fluorescence, suggesting that not all aggressive foci can be reliably detected with this approach (Hosmann et al., 2021). Similarly, Utsuki et al. observed cases where visible 5-ALA fluorescence was absent despite the presence of proliferative activity on histology (Utsuki et al., 2007).These discrepancies might be explained by intertumoral variability in metabolic activity, blood-brain barrier integrity, and PpIX accumulation pathways. In our study, both FS and 5-ALA expression resulted more frequently related to grade 3 gliomas than grade 2 (image c). This relationship was more relevant for 5-ALA (RR = 1.26, CI: 0.95–1.66, p = 0.112). In particular, 5-ALA was positive for 15/60 grade 2 gliomas (25.0 %) and 26/60 grade 3 gliomas (43.3 %). The correlation between increased cell density and fluorescence intensity was also reported by the other studies. Among these, in a study from Widhalm et al. including different grades of gliomas, the lesions with negative ALA fluorescence demonstrated lower MIB-1 labeling and lower PETmax. Moreover, grade 2 gliomas demonstrated lower fluorescence rates compared to grade 3 gliomas, supporting that, with lower features of malignancy, there is less frequent ALA fluorescence labeling (Widhalm et al., 2010). For FS expression, results from our study highlighted a relationship with tumor grade but the statistical significance was not reached (p = 0.055). Even though BBB disruption is more frequent in case of highly proliferating tumors, the link between FS uptake and tumor grade is less linearly represented.

In some previous studies, patients were analyzed for 5-ALA fluorescence related to the clinical outcome in terms of PFS and OS. For example, Jaber et al. described a relationship between positive fluorescence and a shorter time to malignant transformation and a shorter overall survival (Jaber et al., 2019).Also, in the study by Hosmann et al., patients with fluorescing lesions had significantly shorter PFS and OS compared to patients with non-fluorescing tumors (Hosmann et al., 2021). Similarly, in our study we identified a relationship between worse prognosis and fluorescence expression. Actually, the multivariate analysis suggest that fluorophore uptake may correlate with a tumor that presents a worse outcome regardless of other confounding factors, like tumor grade and CE on pMRI. Nevertheless, the comparison of the results for grade 2 and grade 3 consented to observe a tendency for worse prognosis in higher grade, as expected. These results further support the role of ALA FGS in preventing the risk of under grading gliomas. In fact, the choice to discard 5-ALA from surgical practice because of low fluorescence rate in gliomas grade 2 and 3, may limit the identification of anaplastic foci. Therefore, the uptake of 5-ALA or both fluorophores may be more useful in highlighting areas of higher aggressiveness, which need to be further characterized from an anatomopathological perspective or indicate a more aggressive behavior of the tumor as a whole, rather than highlighting the limits of the infiltrated area, unlike how fluorophores are routinely used in HGGs (Hendricks et al., 2019). These findings are confirmed in a recent and comprehensive review by Picart et al., which provides a detailed overview of FGS in LGGs, offering valuable insights into both macroscopic fluorescence and emerging optical imaging technologies (Picart et al., 2024). As also highlighted in our study, the authors report that neither 5-ALA nor FS appear suitable to reliably guide resection or biopsy in LGGs. Regarding 5-ALA, its ability to detect biologically aggressive foci is confirmed, while for FS, evidence in LGGs remains limited—despite it being the preferred fluorophore for CLE. Future research in the context of LGGs should focus on the development of new molecules, such as tozuleristide, and on refining fluorescence detection techniques, including spectroscopy for 5-ALA and CLE for FS (Bianconi et al., 2023; Cossu et al., 2024).

### 4.1 Limitations

Some limitations of the present study need to be addressed. First, the retrospective design of the study, conducted on a highly selected patients’ cohort, does not allow for definitive conclusions about the use of double fluorescence in these tumors. Our cohort reflects a specific subset of patients with WHO grade 2 and 3 gliomas who underwent surgery with the use of intraoperative fluorescence. As such, the present findings should be interpreted within this surgical context.

Not in all MRIs a thin-slice FLAIR sequence was available, allowing for a volumetric analysis of the EOR, particularly for patients who underwent surgery before 2018. Furthermore, the level of fluorescence was classified as negative or positive, without further evaluating the pattern of expression. In fact, we do not dispose of commonly shared criteria to further classify the fluorescence as “faint” or “intense”. Moreover, this assessment would have fragmented the results in a context generally presenting low-fluorescence rate.

## 5 Conclusions

Accordingly with the available literature concerning FGS with FS and 5-ALA, the results achieved in this study do not support the role of these dyes to intraoperatively define the extent of resection, because of low fluorescence rates. Nevertheless, 5-ALA expression could be used to intraoperatively identify more aggressive foci and to supply prognostic information before the histological analysis. Indeed, FS is mostly related to blood-brain barrier damage and, thus, with contrast enhancement in MRI. Anyway, the possible benefits of FGS for grade 2–3 tumors need to be further investigated and established. In fact, many possible strategies may increase the accuracy of tumor intraoperative detection.

## Conflict of interest statement

The authors declare that there are no financial or non-financial conflicts of interest related to the content of this manuscript. None of the authors have received any funding, grants, or benefits from commercial, academic, or other institutions that could influence the outcomes or interpretations of this work.
